# Supplementary material for: Cofilactin filaments regulate filopodial structure and dynamics in neuronal growth cones
Source: Nat Commun. 2022 May 4;13:2439. doi: 10.1038/s41467-022-30116-x (PMC9068697; doi:10.1038/s41467-022-30116-x)
Supplement: Supplementary file 1 — Supplementary Information [file 41467_2022_30116_MOESM1_ESM.docx]

**Supplementary Notes:**

Permeabilization Methods for Immunofluorescence of Growth Cone Cofilactin Bundles (Figs. 1 and Supplementary Figure 4):

**Figure 1 and Supplementary Figure 4** contain immunofluorescence microscopy images that show cofilactin bundles in growth cones. Depending on the additional staining performed (actin or fascin), different fixation and permeabilization protocols were used.

We have observed that the use of Triton-X 100 as a permeabilizing agent before immunolabeling disrupts cofilactin bundle staining (**Supplementary** **Fig. 4A**). Cofilin is still labeled throughout the cell, but there are no discernable aggregates of cofilin signal beneath filopodia. In contrast, the use of organic solvents (ice-cold acetone or methanol) for permeabilization preserves cofilactin bundle staining. Interestingly, this phenomenon has also been shown for cofilin-actin rods ^1–3^.

When staining cofilin and actin, acetone was used as a permeabilizing agent as methanol destroys the phalloidin binding site on actin (**Supplementary** **Fig. 4B**). When staining cofilin and fascin, methanol was used as a fixative instead of PFA (**Supplementary** **Fig. 4C**). Methanol is used since PFA disrupts the binding of our fascin antibody. Methanol acts as a fixative and permeabilizer in this case. Cofilactin bundles also stain well with PFA fixation and methanol permeabilization. We have noticed that acetone slightly diminishes phalloidin and cofilin signal and that cofilin bundle morphology is best preserved with methanol fixation and permeabilization. This is why filopodial bundle length and cofilactin bundle frequency measurements were made on growth cones treated in this way.

Definition of the Filopodial “Transition Region” (**Supplementary Fig. 1**):

For colocalization analysis (**Supplementary** **Fig. 1**), either an entire filopodium was measured, or, if measuring the transition region, a rectangular box that ran 400-nm along the length of the filopodia and was equal to their width was placed in the middle of the filopodia where, on the merged image with both channels activated, the fascin and cofilin signals met. The box was placed immediately distal (towards the fascin-labelled portion) to this spot. This location is diagrammed in **Figure 1E**. The transition region is also referred to as an inflection point throughout this manuscript.

**Supplementary References:**

1. Bamburg, J. R. *et al.* ADF/Cofilin-actin rods in neurodegenerative diseases. *Curr Alzheimer Res* 7, 241–250 (2010).

2. Bernstein, B. W., Shaw, A. E., Minamide, L. S., Pak, C. W. & Bamburg, J. R. Incorporation of cofilin into rods depends on disulfide intermolecular bonds: implications for actin regulation and neurodegenerative disease. *J. Neurosci.* 32, 6670–6681 (2012).

3. Minamide, L. S. *et al.* Isolation and characterization of cytoplasmic cofilin-actin rods. *Journal of Biological Chemistry* 285, 5450–5460 (2010).

**
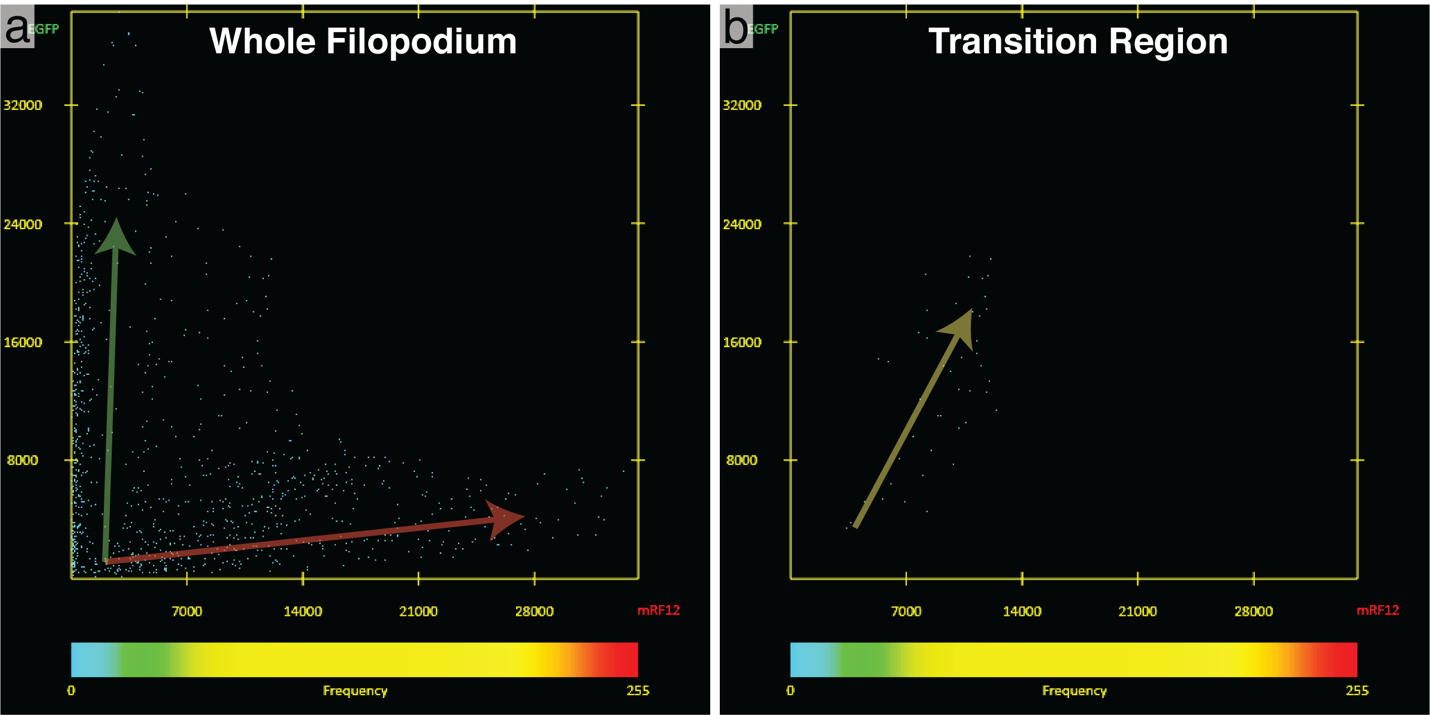
Supplementary** **Fig. 1.**

**Colocalization of fascin and cofilin in neuronal growth cone filopodia.** **(a) & (b)** Representative scattergrams resulting from colocalization analysis of fascin and cofilin in a whole filopodium (a) and the transition region (b) (as defined in the Supplementary Notes and shown in Figure 1E). The average Pearson’s Correlation Coefficient for whole filopodia was -0.28 +/- 0.19 (+/- S.D.) and for the transition region was 0.39 +/- 0.29. Green and red arrows in (a) show pixels that predominately had fascin or cofilin signal, respectively. The yellow arrow in (b) brings attention to the linear relationship between pixel intensities in the green and red channels in the transition region.


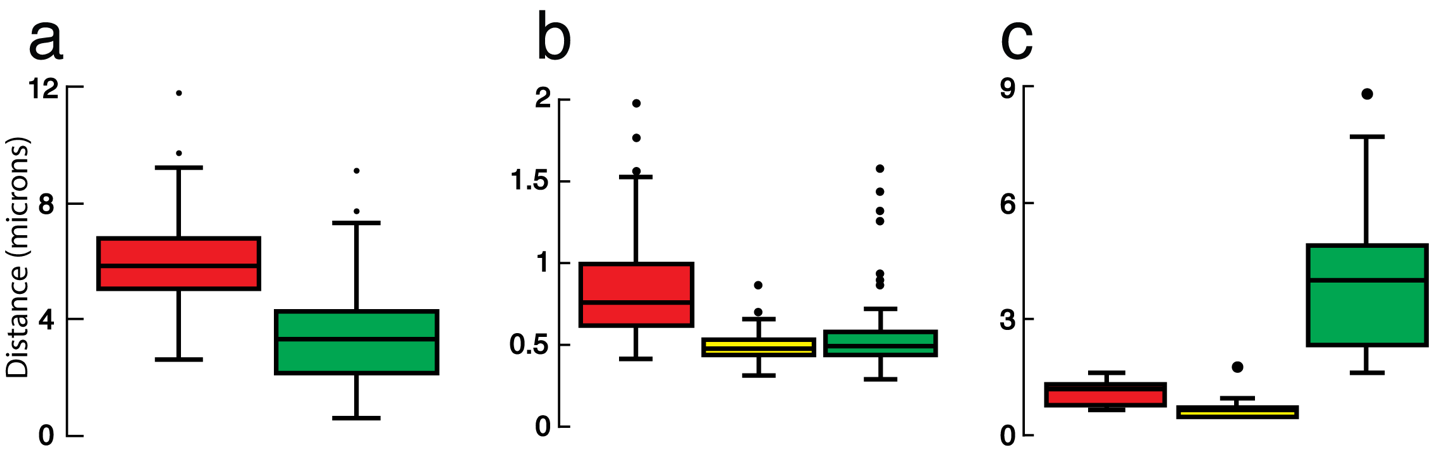


**Supplementary** **Fig. 2.**

**Box plots of data from throughout the paper.** **(a)** Box plot showing the average length of the fascin-labeled portion of filopodia with (red, n = 97) and without (green, n = 72) a cofilactin bundle at their base. Graphs in **(b)** and **(c)** show the average width of cofilin (red), actin (green), and the inflection points (yellow) within MIPS made from live-cell movies of resting ((b), n = 64) and searching ((c), n = 15) filopodia. The y-axis values for all plots are in µm. The middle line in each box represents the median. The top and bottom of each box represent the third and first quartile measurements, respectively. Error bars show the range of the data without outliers. Outliers were designated as such using the interquartile range method. Source data are provided as a Source Data file.


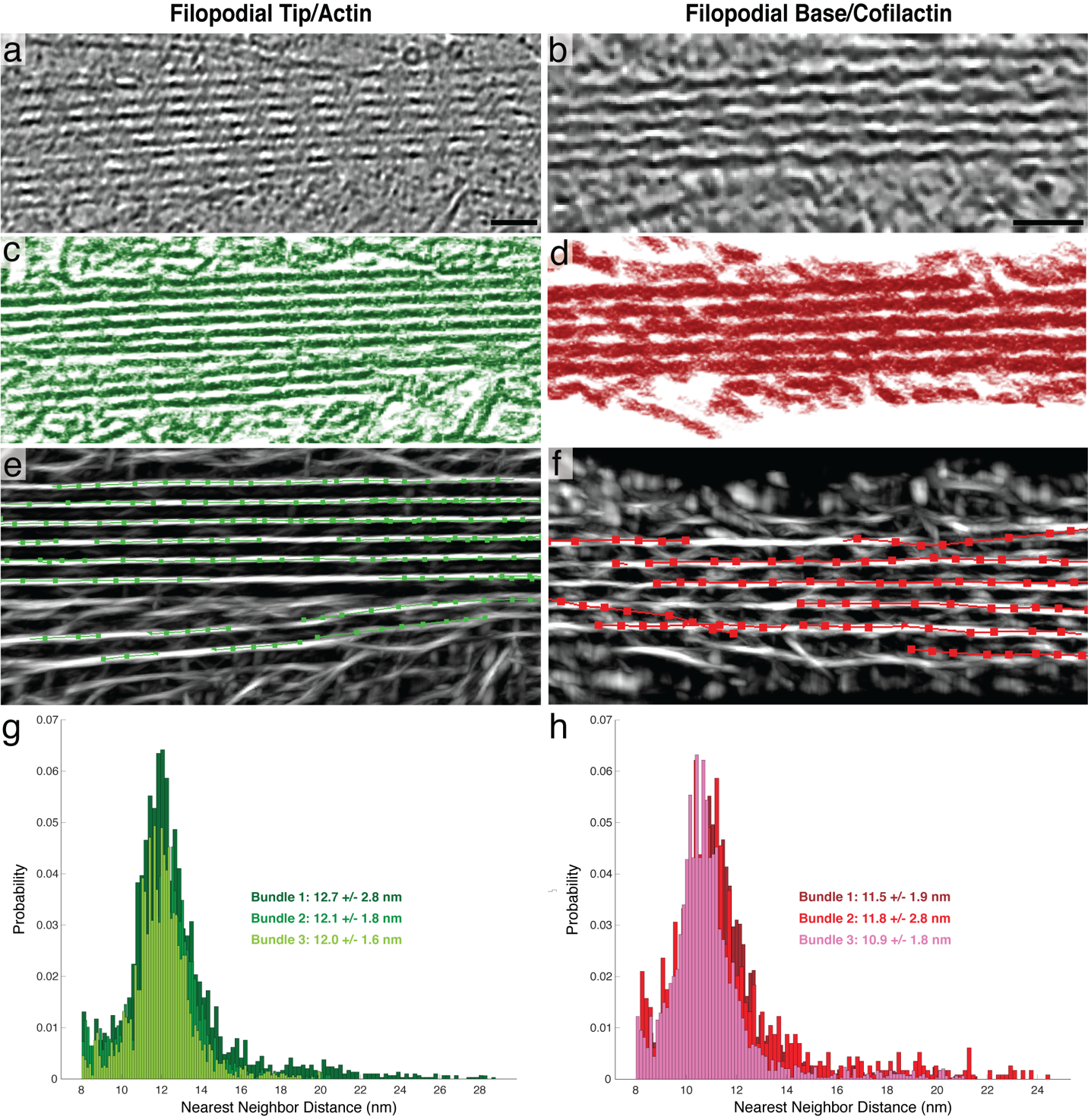


**Supplementary** **Fig. 3.**

**Neural network segmentation and interfilament distance analysis workflow.** **(a-f)** The workflow for the segmentation and filament centerline extraction is displayed in the following way (filopodial tips/actin bundles and filopodial bases/cofilactin bundles were analyzed the same as one another): (a) and (b) show example raw tomograms that were segmented. (c) and (d) show annotated actin and cofilactin filaments, respectively, after segmentation in Dragonfly. (e) and (f) show centerlines that were extracted from filaments using Amira. The points along each line were the points from which nearest neighbor calculations were made in MATLAB using a custom script. **(g & h)** Three bundles of each type were segmented and analyzed. Nearest neighbor measurements are plotted as histograms and all bundles from each type are overlaid together. Bundles from each type overlap heavily, indicating a consistent structure across multiple filopodia. Scale bars in (a) and (b) are 40 nm.


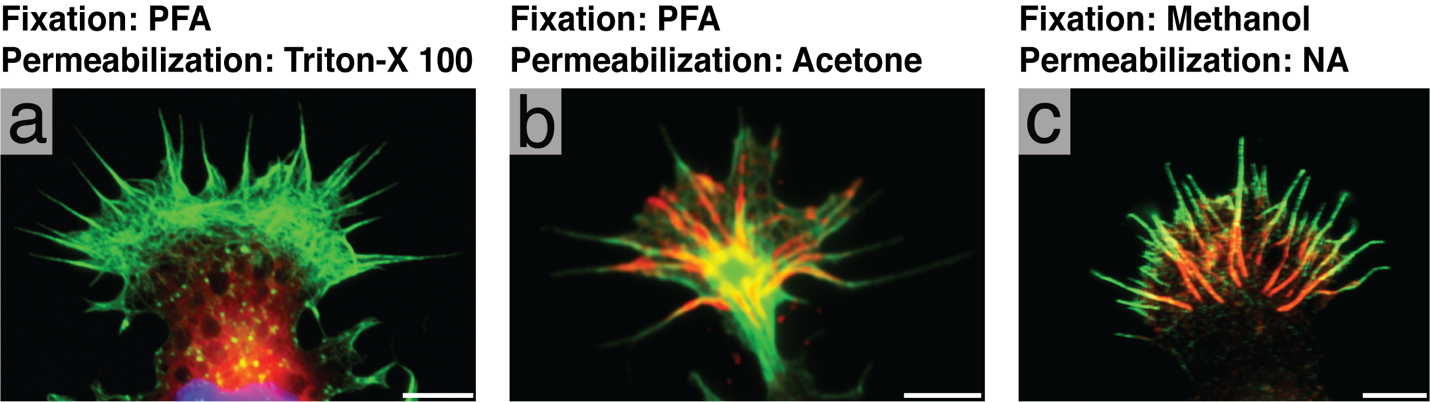


**Supplementary** **Fig. 4.**

**Effect of permeabilization on cofilactin bundle visualization.** As discussed in the Supplementary Notes, multiple fixation and permeabilization protocols were used prior to immunolabeling growth cones. In general, permeabilization with Triton-X 100 seems to prohibit cofilactin bundle labeling, while permeabilization with organic solvents does not. In (a) and (b), green is actin (phalloidin) and red is cofilin. In (c) green is fascin and red is cofilin. **(a)** Fixation with 4% PFA followed by permeabilization with 0.5% Triton-X 100 enables cofilin labeling, but no cofilactin bundles are visible. **(b)** Fixation with 4% PFA and permeabilization with ice-cold acetone preserves cofilactin bundle morphology, although slightly reduces phalloidin and cofilin signal intensity. **(c)** Fixation with methanol preserves cofilactin bundle morphology but destroys phalloidin’s binding epitope, so fascin is used to label the more distal filopodial regions. Methanol also acts as a permeabilizing agent. (a) and (b) are representative images from two independent experiments and (c) is a representative image from three independent experiments. Scale bars are all 5 µm.


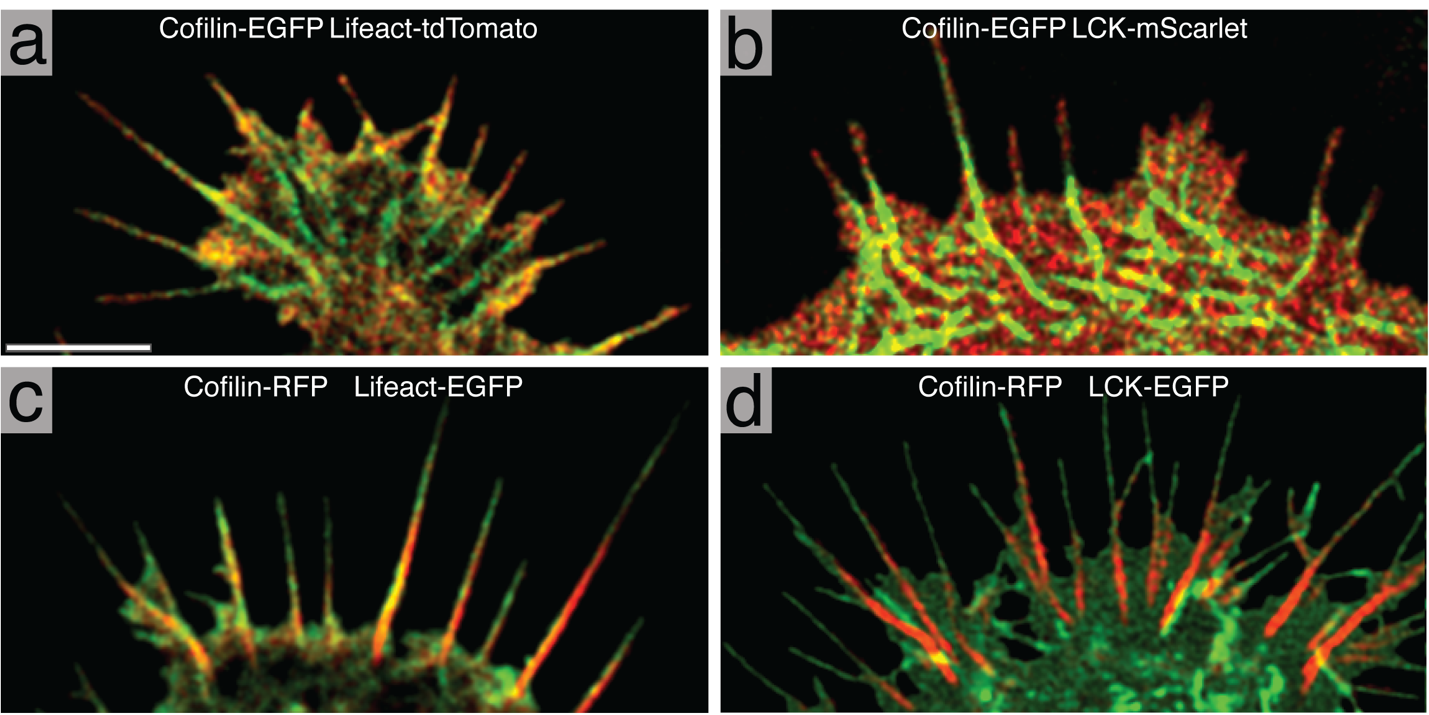


**Supplementary** **Fig. 5.**

**A cofilin-rich filopodial base is observed with numerous pairs of fluorescent tags.** Cofilactin bundles were observed at the base of growth cone filopodia in neurons transfected with both EGFP-cofilin and RFP-cofilin. Moreover, they were also seen when either Lifeact (a small peptide that binds to F-actin) or LCK (a membrane marker) were used in conjunction with cofilin. **(a)** Cofilin-EGFP and Lifeact-tdTomato, **(b)** Cofilin-EGFP and LCK-mScarlet, **(c)** Cofilin-RFP and Lifeact-EGFP, **(d)** Cofilin-RFP and LCK-EGFP. Similar results were observed in multiple cells from two independent experiments for (a), one experiment for (b), three experiments for (c), and four experiments for (d). Scale bar in (a) is 5 µm and also corresponds to (b)-(d).
