# Supplementary material for: Cofilactin filaments regulate filopodial structure and dynamics in neuronal growth cones
Source: Nat Commun. 2022 May 4;13:2439. doi: 10.1038/s41467-022-30116-x (PMC9068697; doi:10.1038/s41467-022-30116-x)
Supplement: Supplementary file 3 — Description of additional Supplementary File [file 41467_2022_30116_MOESM3_ESM.pdf]

Descriptions of Additional Supplementary files

Supplementary Movie 1. Cryo-tomogram of a growth cone filopodium. Tomographic reconstruction of a growth cone filopodial protrusion. Tightly bundled actin filaments nearly fill the entire cytoplasmic space, and fascin crosslinking densities can be seen at regular intervals along the bundle. This movie is of the region depicted in Figure 2B.

Supplementary Movie 2. Cryo-tomogram near a growth cone filopodium's base. Tomographic reconstruction of a growth cone filopodial base. Individual actin filaments surround a bundle of hyper-twisted cofilactin filaments. This movie is of the region depicted in Figure 2C.

Supplementary Movie 3. Cryo-tomogram of a prospective filopodial transition region. Tomographic reconstruction of the region depicted in Figure 5A.

Supplementary Movie 4. Cryo-tomogram of another prospective filopodial transition region. Tomographic reconstruction of the region depicted in Figure 5B.

Supplementary Movie 5. Cryo-tomogram of a cofilactin bundle in the central domain of a growth cone, near the neurite shaft. Tomographic reconstruction of the region depicted in Figure 5C.

Supplementary Movie 6. Movie of whole cell co-expressing fluorescent cofilin and Lifeact. Neurons were transfected with pEGFP cofilin (red) and tdTomato Lifeact (green). The movie is pseudocolored such that Lifeact is green and cofilin is red to match the color scheme used throughout this study. Images were taken every 3 seconds for 10 minutes. This is the same cell as in Figure 6A.

Supplementary Movie 7. Breakage of filopodium in the cofilactin bundle region. Zoomed in movie of filopodia depicted in Figure 6D.

Supplementary Movie 8. Cofilactin bundle "wave" during filopodial motion. Zoomed in movie of filopodia depicted in Figure 6E.

Supplementary Movie 9. Movie of filopodial bending. Zoomed in movie of filopodia depicted in Figure 6F. S

Supplementary Movie 10. Cryo-tomogram at a kink in a filopodium. Tomographic reconstruction of the region depicted in Figure 7A.

Supplementary Movie 11. Cryo-tomogram of a filopodial "wave". Tomographic reconstruction of the region depicted in Figure 7B. Supplementary Movie 12. Cryo-tomogram at a bend in a filopodium. Tomographic reconstruction of the region depicted in Figure 7C.
