# Supplementary material for: Cofilactin filaments regulate filopodial structure and dynamics in neuronal growth cones
Source: Nat Commun. 2022 May 4;13:2439. doi: 10.1038/s41467-022-30116-x (PMC9068697; doi:10.1038/s41467-022-30116-x)
Supplement: Supplementary file 16 — Reporting Summary [file 41467_2022_30116_MOESM16_ESM.pdf]

nature portfolio

Corresponding author(s): Matthew Swulius

Last updated by author(s): Mar 24, 2022

Reporting Summary

Nature Portfolio wishes to improve the reproducibility of the work that we publish. This form provides structure for consistency and transparency in reporting. For further information on Nature Portfolio policies, see our Editorial Policies and the Editorial Policy Checklist.

Statistics

For all statistical analyses, confirm that the following items are present in the figure legend, table legend, main text, or Methods section.

n/a	Confirmed	
☐	☒	The exact sample size (*n*) for each experimental group/condition, given as a discrete number and unit of measurement
☐	☒	A statement on whether measurements were taken from distinct samples or whether the same sample was measured repeatedly
☒	☐	The statistical test(s) used AND whether they are one- or two-sided<br>*Only common tests should be described solely by name; describe more complex techniques in the Methods section.*
☒	☐	A description of all covariates tested
☐	☒	A description of any assumptions or corrections, such as tests of normality and adjustment for multiple comparisons
☐	☒	A full description of the statistical parameters including central tendency (e.g. means) or other basic estimates (e.g. regression coefficient) AND variation (e.g. standard deviation) or associated estimates of uncertainty (e.g. confidence intervals)
☒	☐	For null hypothesis testing, the test statistic (e.g. *F*, *t*, *r*) with confidence intervals, effect sizes, degrees of freedom and *P* value noted<br>*Give P values as exact values whenever suitable.*
☒	☐	For Bayesian analysis, information on the choice of priors and Markov chain Monte Carlo settings
☒	☐	For hierarchical and complex designs, identification of the appropriate level for tests and full reporting of outcomes
☐	☒	Estimates of effect sizes (e.g. Cohen's *d*, Pearson's *r*), indicating how they were calculated

Our web collection on statistics for biologists contains articles on many of the points above.

Software and code

Policy information about availability of computer code

Data collection	Zeiss Zen Blue version 3.1, FEI Tomography version 4
Data analysis	FIJI version 2.1.0/1.53(, Zeiss Zen Blue version 3.1, IMOD version 4.11, PEET version 1.14, Dynamo version 1.1.514, Dragonfly version 2021.1, Amira version 6.3, UCSF Chimera version 1.13, MATLAB 2021a

For manuscripts utilizing custom algorithms or software that are central to the research but not yet described in published literature, software must be made available to editors and reviewers. We strongly encourage code deposition in a community repository (e.g. GitHub). See the Nature Portfolio guidelines for submitting code & software for further information.

Data

Policy information about availability of data

All manuscripts must include a data availability statement. This statement should provide the following information, where applicable:

- Accession codes, unique identifiers, or web links for publicly available datasets
- A description of any restrictions on data availability
- For clinical datasets or third party data, please ensure that the statement adheres to our policy

The subtomogram averaging data that support the findings of this study are available on the Electron Microscopy Data Bank (EMDB) under the accession codes EMD-26214 [https://www.ebi.ac.uk/emdb/EMD-26214] (actin average) and EMD-26215 [https://www.ebi.ac.uk/emdb/EMD-26215] (cofilactin average). Source data are provided as a Source Data file.

Field-specific reporting

Please select the one below that is the best fit for your research. If you are not sure, read the appropriate sections before making your selection.

- [x] Life sciences
- [ ] Behavioural & social sciences
- [ ] Ecological, evolutionary & environmental sciences

For a reference copy of the document with all sections, see nature.com/documents/nr-reporting-summary-flat.pdf

Life sciences study design

All studies must disclose on these points even when the disclosure is negative.

Sample size	Sample size was not determined prior to data collection, because we characterized observations of wildtype protein distritubtion only (no comparison between experimental groups). Sample sizes were determined for live-cell imaging and tomography by the number of growth cones and filopodia present for imaging. For immunofluorescence, a minimum of three filopodia from three growth cones on three different cells were measured. We considered this sufficient, because we are only characterizing wildtype distrubution and did not see any difference between replicates.
Data exclusions	In the interfilament distance analysis data points more than 3 standard deviations from the mean were excluded. The rationale behind this is that some of the filaments in the tomograms were not part of the bundle of interest and provided anomalous measurements. Data exclusion was not predetermined. It was only used during the interfilament distance measurements, and was empirically determined based on feedback from the analysis. Some filaments were simply not part of the bundle and showed up as outliers.
Replication	All experiments were done in duplicates or triplicates, and all attempts were successful.
Randomization	We did not randomize data collection, because we were only characterizing wildtype cell.
Blinding	No blinding was used, because we were only characterizing wildtype cells.

Reporting for specific materials, systems and methods

We require information from authors about some types of materials, experimental systems and methods used in many studies. Here, indicate whether each material, system or method listed is relevant to your study. If you are not sure if a list item applies to your research, read the appropriate section before selecting a response.

Materials & experimental systems

n/a	Involved in the study	
☐	☒	Antibodies
☒	☐	Eukaryotic cell lines
☒	☐	Palaeontology and archaeology
☒	☐	Animals and other organisms
☒	☐	Human research participants
☒	☐	Clinical data
☒	☐	Dual use research of concern

Methods

n/a	Involved in the study	
☒	☐	ChIP-seq
☒	☐	Flow cytometry
☒	☐	MRI-based neuroimaging

Antibodies

Antibodies used	1. Anti-Fascin 1 Antibody (from mouse), Santa Cruz Biotechnology, Catalog Number: sc-21743, Lot Number: E2014. Dilution 1:500.<br>2. Anti-Cofilin Antibody (from rabbit, Sigma-Aldrich, Catalog Number: C8736, Lot Number: multiple used. Dilution 1:1000.<br>3. Goat anti-Mouse Alexa Fluor 488, Abeam, Catalog Number: ab150117, Lot Number: multiple used. Dilution 1:500.<br>4. Goat anti-Rabbit Alexa Fluor 594, Abeam, Catalog Number: ab 150080, Lot Number: multiple used. Dilution 1:500.
Validation	1. Anti-Fascin 1 Antibody: validation provided by manufacturer (https://www.scbt.com/p/fascin-1-antibody-55k-2)<br>2. Anti-Cofilin Antibody: validation provided by manufacturer (https://www.sigmaaldrich.com/US/en/product/sigma/c8736)
